# Monomyristin and Monopalmitin Derivatives: Synthesis and Evaluation as Potential Antibacterial and Antifungal Agents

**DOI:** 10.3390/molecules23123141

**Published:** 2018-11-29

**Authors:** Jumina, Asma Nurmala, Anggit Fitria, Deni Pranowo, Eti Nurwening Sholikhah, Yehezkiel Steven Kurniawan, Bambang Kuswandi

**Affiliations:** 1 Department of Chemistry, Faculty of Mathematics and Natural Sciences, Universitas Gadjah Mada, Sekip Utara, Yogyakarta 55281, Indonesia; asma_nurmala@yahoo.co.id (A.N.); anggitfitria@gmail.com (A.F.); maspranowo@ugm.ac.id (D.P.); kurniawan94steven@gmail.com (Y.S.K.); 2 Department of Pharmacology and Therapy, Faculty of Medicine, Universitas Gadjah Mada, Sekip Utara, Yogyakarta 55281, Indonesia; etinurweningsholikhah@ugm.ac.id; 3 Faculty of Pharmacy, University of Jember, Jember 68121, Indonesia; b_kuswandi.farmasi@unej.ac.id

**Keywords:** monomyristin, monopalmitin, synthesis, antibacterial, antifungal

## Abstract

In the present work, monoacylglycerol derivatives, i.e., 1-monomyristin, 2-monomyristin, and 2-monopalmitin were successfully prepared from commercially available myristic acid and palmitic acid. The 1-monomyristin compound was prepared through a transesterification reaction between ethyl myristate and 1,2-*O*-isopropylidene glycerol, which was obtained from the protection of glycerol with acetone, then followed by deprotection using Amberlyst-15. On the other hand, 2-monoacylglycerol derivatives were prepared through enzymatic hydrolysis of triglycerides in the presence of *Thermomyces lanuginosa* lipase enzymes. The synthesized products were analyzed using fourier transform infrared (FTIR) spectrophotometer, gas or liquid chromatography-mass spectrometer (GC-MS or LC-MS), and proton and carbon nuclear magnetic resonance (^1^H- and ^13^C-NMR) spectrometers. It was found that monomyristin showed high antibacterial and antifungal activities, while 2-monopalmitin did not show any activity at all. The 1-monomyristin compound showed higher antibacterial activity against *Staphylococcus aureus* and *Aggregatibacter actinomycetemcomitans* and also higher antifungal activity against *Candida albicans* compared to the positive control. Meanwhile, 2-monomyristin showed high antibacterial activity against *Escherichia coli*. The effect of the acyl position and carbon chains towards antibacterial and antifungal activities was discussed.

## 1. Introduction

Harmful microorganisms cause serious problems in industrial food products. One of the gram-negative bacteria, *Escherichia coli* (*E. coli*), has been reported for serious food poisoning and diarrhea [1]. *Staphylococcus aureus* (*S. aureus*), one of the gram-positive bacteria, causes skin infections [2], while *Candida albicans* (*C. albicans*) gives rise to nosocomial and superficial infections [3]. Many researchers have been putting effort into developing an effective and efficient antibacterial and antifungal agent from natural product isolation [4,5,6]. He et al. (2017) reported polyketide derivatives from *Emericella* sp. TJ29 and evaluated their antibacterial activity [7]. However, isolation from natural resources has several disadvantages, such as time-consuming process, low-isolation yield, high price, limited resources, and disturbance of food and environmental sustainability [8]. Because of this, researchers prefer to develop synthetic compounds as antibacterial and antifungal agents [9,10,11,12].

Fatty acids and monoacylglycerol derivatives are widely developed as antibacterial agents [13]. Monoacylglycerol can be prepared through selective glycerolysis by using an inorganic base catalyst under a nitrogen atmosphere [14]. Lauric acid and monocaprin have been investigated as intravaginal microbicides to correct sexually transmitted diseases [15,16]. In our previous work, the 1-monolaurin compound was prepared in a simple and efficient method from lauric acid and glycerol in the presence of *p*-toluenesulfonic acid as the catalyst [17,18]. Chinatangkul et al. (2018) reported that monolaurin compounds can be loaded onto shellac nanofibers and used as an antimicrobial agent [19]. Even though monolaurin derivatives have been well investigated, only few reports are found for the synthesis and evaluation of other monoacylglycerol derivatives, such as monomyristin and monopalmitin.

In this work, we prepared other derivatives of monoacylglycerol, i.e., 1-monomyristin, 2-monomyristin, and 2-monopalmitin, from myristic acid and palmitic acid. The 1-monomyristin derivative was synthesized through esterification of myristic acid with ethanol and further reacted with 1,2-*O*-isopropylidene glycerol, then followed by hydrolysis using Amberlyst-15 as a solid acid catalyst. The 2-monoacylglycerol derivatives were initially produced via esterification of glycerol with ethyl myristate to form triglyceride, followed by selective hydrolysis of the triglyceride using *Thermomyces lanuginosa* lipase enzymes (TLIM).

## 2. Results and Discussion

### 2.1. Synthesis of 1-Monomyristin

**Figure 1:**
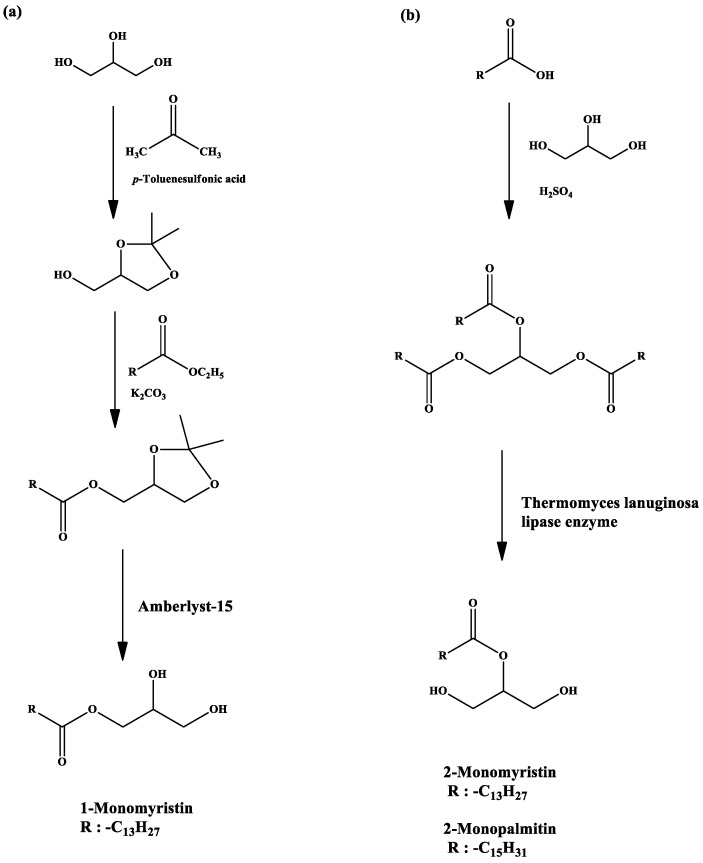
The synthesis scheme of (**a**) 1-monoacylglycerol and (**b**) 2-monoacylglycerol derivatives.

The synthesis of the 1-monomyristin compound consisted of four steps, i.e., synthesis of 1,2-*O*-isopropylidene glycerol and ethyl myristate separately, synthesis of isopropylidene glycerol myristate, and synthesis of 1-monomyristin (Figure 1a). Selective monosubstitution of glycerol on position 1 can be obtained after the protection of the two hydroxyl groups of glycerol by using acetone. In this work, glycerol was reacted with acetone in the presence of *p*-toluenesulfonic acid (*p*TSA) as an acid catalyst to obtain a ketal derivative of glycerol, i.e., 1,2-*O*-isopropylidene glycerol. The 1,2-*O*-isopropylidene glycerol was then transesterified with ethyl myristate in basic conditions to obtain isopropylidene glycerol myristate. Finally, the isopropylidene glycerol myristate was deprotected using Amberlyst-15. The chemical structure of the intermediates was confirmed from the FTIR and GC-MS analysis. The 1-monomyristin product was successfully synthesized in this work in a quantitative yield (100%) by a simple stirring method with small amount (0.04 g) of Amberlyst-15 as heterogeneous catalyst. The FTIR spectra of 1-monomyristin show a broad peak at 3456 cm^−1^ and a strong peak at 1735 cm^−1^ due to the presence of hydroxyl and carbonyl groups, respectively. The purity of the product was confirmed from LC chromatograms and the mass spectrum show [M + H]^+^ at *m*/*z* = 303. The ^1^H- and ^13^C-NMR spectra of 1-monomyristin are shown in Figures S1 and S2, respectively. Both ^1^H- and ^13^C-NMR spectra confirmed the formation of 1-monomyristin product. The presence of the hydroxyl groups of the 1-monomyristin product is shown as singlet peak at 2.18 ppm, while the protons of the glycerol are observed at chemical shifts more than 3.50 ppm because the carbons were directly bonded to oxygen atoms. The ^13^C-NMR spectra also show 3 peaks at 63.35, 65.16 and 70.28 for the inequivalent carbon atoms in the glycerol backbone.

### 2.2. Synthesis of 2-Monomyristin and 2-Monopalmitin

The synthesis of 2-monomyristin was carried out through trimyristin synthesis and followed by enzymatic hydrolysis with TLIM as catalyst (Figure 1b). Trimyristin was prepared by an esterification reaction between glycerol and myristic acid under acidic conditions. The excess of myristic acid was removed in basic conditions because sodium myristate is soluble in water. The product remained in the organic phase. The chemical structure of trimyristin product was confirmed by FTIR, GC-MS, and ^1^H- and ^13^C-NMR analysis. The absence of the broad peak at 3200–3400 cm^−1^ on FTIR spectra shows that all the hydroxyl groups of the glycerol were completely esterified with myristic acid. The purity of the product was confirmed from GC chromatograms while the mass spectrum corresponds to the trimyristin fragmentation. Both ^1^H- and ^13^C-NMR spectra confirmed the absence of hydroxyl proton and the presence of carbon of C=O ester at 173.54 ppm, respectively. Afterwards, the myristate esters at the edge of the trimyristin were selectively hydrolyzed in the presence of TLIM as the catalyst. The 2-monomyristin compound was purified through preparative thin layer chromatography (PTLC) and its chemical structure was confirmed by FTIR, GC-MS, and ^1^H- and ^13^C-NMR analysis. A broad peak appeared due to the presence of hydroxyl groups of 2-monomyristin. The purity of the product was confirmed from LC chromatograms and the mass spectrum corresponds to the 2-monomyristin fragmentation. The ^1^H- and ^13^C-NMR spectra of 2-monomyristin are shown in Figures S3 and S4, respectively. Both ^1^H- and ^13^C-NMR spectra confirmed the formation of 2-monomyristin product.

The synthesis process of 2-monopalmitin is similar to the synthesis of 2-monomyristin (Figure 1b). Tripalmitin was prepared from an esterification reaction and then hydrolyzed in the presence of TLIM as the catalyst to obtain 2-monopalmitin as the final product. The chemical structure of both tripalmitin and 2-monopalmitin was confirmed by FTIR, GC-MS, and ^1^H- and ^13^C-NMR analysis. The absence of the broad peak at 3200–3400 cm^−1^ on tripalmitin FTIR spectrum shows that all the hydroxyl groups of the glycerol were completely esterified with palmitic acid. The purity of the tripalmitin product was confirmed from the presence of a single peak from the GC chromatogram, and its mass spectrum is in agreement with the expected fragmentation of tripalmitin compound. Both ^1^H- and ^13^C-NMR spectra confirmed the absence of hydroxyl proton and the presence of the C=O ester, respectively. On the other side, a broad peak appeared due to the presence of the hydroxyl groups of 2-monopalmitin. The purity of the product was confirmed from LC chromatograms, and the structure of the product was further confirmed by the mass spectra and ^1^H- and ^13^C-NMR. The ^1^H- and ^13^C-NMR spectra of 2-monopalmitin are shown in Figures S5 and S6, respectively.

### 2.3. Antibacterial and Antifungal Assays of Products

The antibacterial and antifungal activities of each synthesized product were evaluated against *E. coli*, *S. aureus,* and *C. albicans*. The result of the biological assay is shown in Table 1. Bold values were made when the inhibition zone of the sample is larger than that of the positive control. It was found that 0.25% 2-monomyristin exhibited higher antibacterial activity than the positive control (1.00% 4-isopropyl-3-methylphenol) against *E. coli*. It was also found that 0.50% 1-monomyristin and 0.50% 2-monomyristin showed higher antibacterial activity compared to the positive control against *S. aureus*. However, only 1-monomyristin showed antifungal activity against *C. albicans*. Since 1-monomyristin showed promising antibacterial and antifungal activity, 1-monomyristin was further investigated as an antibacterial agent against *Bacillus subtilis* (*B. subtilis*) and *Aggregatibacter actinomycetemcomitans* (*A. actinomycetemcomitans*). The antibacterial activity of 1-monomyristin against *B. subtilis* and *A. actinomycetemcomitans* is listed in Table 2. As expected, 1-monomyristin exhibited antibacterial activity against *B. subtilis* and *A. actinomycetemcomitans*. From these results, monomyristin showed better antibacterial and antifungal activities compared to monopalmitin. This is probably due to the shorter carbon chain near the monolauric acid [18]. The 1-position showed high activity for both antibacterial and antifungal agent. It is due to the better interaction 1-monomyristin can make with the bacterial cell wall than 2-monomyristin can.

The inhibition mechanism of 1-monomyristin against *C. albicans* seems to be similar to amphotericin B compounds. The hydroxyl group in 1-monomyristin interacts with the ergosterol on the fungi cell membrane, and therefore the function of the membrane was disrupted and cell lysis happened [20]. In contrast to the *C. albicans* cell wall, which consists of the phospholipid bilayer, ergosterol, chitin, and microfibrillar *β*-glucan, the bacterial cell wall is simpler. Therefore, it is reasonable for monomyristin compounds that showed high activity to be used as antibacterial agents against *E. coli*, *S. aureus*, *B. subtilis*, and *A. actinomycetemcomitans*. The 1-monomyristin compound at concentrations higher than 1.00% exhibited medium antifungal activity compared with 1.00% 4-isopropyl-3-methylphenol as the positive control. These results demonstrate that 1-monomyristin is a potential compound to be applied as an antibacterial and antifungal agent.

## 3. Materials and Methods

### 3.1. Materials

Myristic acid, palmitic acid, glycerol, sulfuric acid, *n*-hexane, ethanol, acetone, sodium hydroxide, *p*-toluenesulfonic acid (*p*TSA), Amberlyst-15, sodium carbonate, potassium carbonate, ethyl acetate, chloroform, and anhydrous sodium sulfate were purchased from Merck (Darmstadt, Germany), while TLIM was obtained from Novozymes (Bagsværd, Denmark). All chemicals were used as received without any further purification. Thin layer chromatography (TLC) was carried out using an aluminium plate (Merck) coated with silica gel 60 F254 (20 × 20 cm). The brain-heart broth and Sabouraud agar powder which contains 4.00% of dextrose as the microbial nutrient was purchased from Merck.

### 3.2. Equipment

Infrared spectra of the prepared compounds were recorded on a Fourier transform infrared spectrophotometer (FT-IR, Shimadzu Prestige 21, Tokyo, Japan). The purity of the products was evaluated from chromatograms which were obtained from either gas chromatography-mass spectrometer (GC-MS, Shimadzu QP 2010S) or liquid chromatography-mass spectrometer (LC-MS/MS, Acquity HPLC-SQD MassLynx v4-1 SCN 805, Waters Corporation, Milford, MA, USA). The ^1^H-NMR (nuclear magnetic resonance) and ^13^C-NMR spectra were recorded by a JEOL JNM–ECZ500R/S1 spectrometer (JEOL Ltd., Tokyo, Japan) using tetramethylsilane as an internal standard in deuterated chloroform.

### 3.3. Synthesis of 1-Monomyristin

#### 3.3.1. Synthesis of 2,2-Dimethyl-1,3-dioxolan-4-methanol (1,2-*O*-isopropylidene glycerol)

Acetone (48 mL, 650 mmol) and *p*TSA (1.2 g) were stirred for 15 min. Chloroform (96 mL) was added into the solution and stirred at 343 K for 30 min. Glycerol (24 mL, 330 mmol) was then added and the mixture was heated at 393 K for 6.5 h. The lower layer was neutralized with sodium carbonate and then dried over anhydrous sodium sulfate. The solvent was evaporated to obtain 14.61 g of 1,2-*O*-isopropylidene glycerol as a colorless liquid (33.71% yield). FT-IR (cm^−1^): 3417 (broad, OH stretching), 2985 (C-H stretching), 1211 (C-O eter). GC: 100% 1,2-*O*-isopropylidene glycerol (retention time (t_R_) = 8.6 min, [M − 15]^+^ = 117).

#### 3.3.2. Synthesis of Ethyl Myristate

Myristic acid (12 g, 53 mmol) was esterified by using a 4% wt sulfuric acid solution in ethanol (50 mL) and the mixture was sonicated for 4.5 h with a Branson 1210 ultrasonic cleaner (Branson, Danbury, CT, USA). After the reaction, the solvent was evaporated. The filtrate was diluted with ethyl acetate (40 mL) and washed with 5% wt sodium hydroxide solution until neutral. The organic layer was dried over anhydrous sodium sulfate and the solvent was evaporated to obtain 13.25 g of ethyl myristate as a yellowish liquid (98.43% yield). FT-IR (cm^−1^): 2924 (C-H stretching), 1735 (C=O ester), 1458 (CH_2_ bending), 1180 (C-O ester). GC: 98.90% ethyl myristate (retention time (t_R_) = 34.4 min, [M − 15]^+^ = 256).

#### 3.3.3. Synthesis of Isopropylidene Glycerol Myristate

The 1-monomyristin compound was prepared in two-step process, i.e., synthesis of the isopropylidene glycerol myristate followed by ketal hydrolysis using Amberlyst-15 (Sigma Aldrich, Missouri, MO, USA). First, ethyl myristate (2.1 g, 8 mmol) was reacted with 1,2-*O*-isopropylidene glycerol (4.2 g, 32 mmol) and potassium carbonate (0.31 g) at 413 K for 30 h. Diethylether (20 mL) was used to extract the product and then neutralized with distilled water. The solvent was evaporated to obtain 0.92 g of isopropylidene glycerol myristate as a yellowish liquid (32.12% yield). FT-IR (cm^−1^): 3441 (broad, OH stretching), 2924 (C-H stretching), 1743 (C=O ester), 1458 (CH_2_ bending), 1157 (C-O ester). GC: 95.55% isopropylidene glycerol myristate (retention time (t_R_) = 30.0 min, [M − 15]^+^ = 327).

#### 3.3.4. Synthesis of 1-Monomyristin

The isopropylidene glycerol myristate (0.4 g, 1 mmol) was directly dissolved in ethanol (5 mL), and the mixture was stirred at room temperature for 30 h in the presence of Amberlyst-15 (0.04 g) as the catalyst. The mixture was filtered and evaporated to obtain 0.31 g of 1-monomyristin as the final product, a white solid (100.00% yield). Melting point: 53.8–56.7 °C. FT-IR (cm^−1^): 3456 (broad, OH stretching), 2916 (C-H stretching), 1735 (C=O ester), 1465 (CH_2_ bending), 1180 (C-O ester). LC: 1-monomyristin (retention time (t_R_) = 3.17 min, [M + H]^+^ = 303). ^1^H-NMR (ppm): 0.87 (t, 3H, -CH_3_), 1.27 (m, 20H, -CH_2_-), 1.63 (q, 2H, -C**H_2_**-CH_2_-CO), 2.18 (s, 2H, OH), 2.35 (t, 2H, -CH_2_-CO), 3.60 (dod, 1H, *J* = 6.0 and 11.5 Hz, -C**H_2_**-OH), 3.70 (dod, 1H, *J* = 6.8 and 10.8 Hz, -C**H_2_**-OH), 3.93 (q, 1H, C**H**-OH), 4.13-4.31 (m, 2H, -C**H_2_**-OOC-). ^13^C-NMR (ppm): 14.14 (-CH_3_), 22.70 (-**C**H_2_-CH_3_), 24.92–31.93 (other -CH_2_-), 34.17 (-**C**H_2_-CH_2_-CO), 34.42 (-**C**H_2_-CO), 63.35 (-CH_2_-OH), 65.16 (-CH_2_-O-), 70.28 (-CH-OH), 174.42 (-COO-).

### 3.4. Synthesis of 2-Monomyristin

#### 3.4.1. Synthesis of Trimyristin

Myristic acid (13.7 g, 60 mmol), glycerol (0.9 g, 10 mmol), and a 5% wt sulfuric acid solution (0.7 mL) were reacted at 393 K for 3 h. Chloroform (96 mL) was added into the solution and stirred at 343 K for 30 min. A 10% wt NaOH solution was added into the mixture until the pH of the mixture reached 13. The product was extracted with n-hexane and the organic layer was washed with distilled water until neutral. The organic phase was dried over anhydrous sodium sulfate to obtain 9.62 g of trimyristin as a yellowish solid (54% yield). Melting point: 56.0–58.0 °C. FT-IR (cm^−1^): 2916 (C-H stretching), 1753 (C=O), 1296 (C-O eter). GC: 100% trimyristin (retention time (t_R_) = 18.9 min, [M − 227]^+^ = 496). ^1^H-NMR (ppm): 0.87 (t, 9H, -CH_3_), 1.27 (m, 30H, -CH_2_-), 1.62 (m, 6H, -C**H_2_**-CH_2_-CO), 2.34 (t, 6H, OH), 2.34 (t, 6H, -CH_2_-CO), 4.28 (dod, 4H, *J* = 4.5 and 11.6 Hz, -C**H_2_**-OOC-), 5.23 (m, 1H, C**H**-OOC-). ^13^C-NMR (ppm): 14.30 (-CH_3_), 22.87 (-**C**H_2_-CH_3_), 24.86–29.85 (other -CH_2_-), 32.10 (-**C**H_2_-CH_2_-CO), 34.17 (-**C**H_2_-CO), 62.29 (-CH_2_-O-), 69.03 (-CH-O-), 173.54 (-COO-).

#### 3.4.2. Synthesis of 2-Monomyristin

The trimyristin (0.75 g, 1 mmol) was directly dissolved in dry ethanol (3 mL), and the mixture was incubated at 308 K for 24 h in the presence of TLIM (0.38 g) as the catalyst. This catalyst was reported for its regioselectivity [21,22]. The mixture was filtered, and the filtrate was diluted with 80% vol ethanol (20 mL). The side products were removed using n-hexane (30 mL). The aqueous phase was evaporated and purified with PTLC with chloroform:acetone:methanol = 9.5:0.45:0.05 as the mobile phase to obtain 0.06 g of 2-myristin (18% yield) as the final product, a yellowish liquid. Melting point: 58.0–60.0 °C. FT-IR (cm^−1^): 3425 (broad, OH stretching), 2924 (C-H stretching), 1735 (C=O ester), 1465 (CH_2_ bending), 1180 (C-O ester). LC: 2-monomyristin (retention time (t_R_) = 0.33 min, [M + H]^+^ = 303). ^1^H-NMR (ppm): 0.85 (t, 3H, -CH_3_), 1.25 (m, 20H, -CH_2_-), 1.58 (m, 2H, -C**H_2_**-CH_2_-CO), 2.33 (t, 2H, -CH_2_-CO), 3.92 (m, 1H, C**H**-OOC-), 4.19 (m, 2H, *J* = 6.5 and 11.5 Hz, -C**H_2_**-OH). ^13^C-NMR (ppm): 14.22 (-CH_3_), 22.78 (-**C**H_2_-CH_3_), 25.00–29.76 (other -CH_2_-), 32.00 (-**C**H_2_-CH_2_-CO), 34.24 (-**C**H_2_-CO), 63.43 and 65.26 (-CH_2_-OH), 70.34 (-CH-OH), 174.63 (-COO-).

### 3.5. Synthesis of 2-Monopalmitin

#### 3.5.1. Synthesis of Tripalmitin

Palmitic acid (15.4 g, 60 mmol), glycerol (0.9 g, 10 mmol), and a 5% wt sulfuric acid solution (0.7 mL) were reacted at 393 K for 3 h. Chloroform (96 mL) was added into the solution and stirred at 343 K for 30 min. 10% wt NaOH solution was added into the mixture until the pH of the mixture reached 13. The product was extracted with *n*-hexane and the organic layer was washed with distilled water until neutral. The organic phase was dried over anhydrous sodium sulfate to obtain 9.62 g of tripalmitin as a yellowish solid (53% yield). Melting point: 65.0–68.0 °C. FT-IR (cm^−1^): 2916 (C-H stretching), 1705 (C=O), 1180 (C-O eter). GC: 100% tripalmitin (retention time (t_R_) = 21.9 min, [M − 567]^+^ = 239). ^1^H-NMR (ppm): 0.87 (t, 9H, -CH_3_), 1.29 (m, 30H, -CH_2_-), 1.62 (m, 6H, -C**H_2_**-CH_2_-CO), 2.34 (t, 6H, OH), 2.34 (t, 6H, -CH_2_-CO), 4.27 (dod, 4H, *J* = 4.5 and 11.6 Hz, -C**H_2_**-OOC-), 5.26 (m, 1H, C**H**-OOC-). ^13^C-NMR (ppm): 14.30 (-CH_3_), 22.88 (-**C**H_2_-CH_3_), 24.86–29.85 (other -CH_2_-), 32.10 (-**C**H_2_-CH_2_-CO), 34.17 (-**C**H_2_-CO), 62.29 (-CH_2_-O-), 69.03 (-CH-O-), 173.54 (-COO-).

#### 3.5.2. Synthesis of 2-Monopalmitin

The tripalmitin (0.75 g, 1 mmol) was directly dissolved in dry ethanol (3 mL), and the mixture was incubated at 308 K for 24 h in the presence of TLIM (0.38 g) as catalyst. The mixture was filtered and the filtrate was diluted with 80%vol ethanol (20 mL). The side products were removed by using n-hexane (30 mL). The aqueous phase was evaporated and purified with PTLC with chloroform:acetone:methanol = 9.5:0.45:0.05 as the mobile phase to obtain 0.03 g of 2-palmitin (8% yield) as the final product, a yellowish solid. Melting point: 67.0–70.0 °C. FT-IR (cm^−1^): 3433 (broad, OH stretching), 2916 (C-H stretching), 1735 (C=O ester), 1465 (CH_2_ bending), 1180 (C-O ester). LC: 2-monomyristin (retention time (t_R_) = 0.32 min, [M]^+^ = 330). ^1^H-NMR (ppm): 0.85 (t, 3H, -CH_3_), 1.25 (m, 20H, -CH_2_-), 1.58 (m, 2H, -C**H_2_**-CH_2_-CO), 2.33 (t, 2H, -CH_2_-CO), 3.92 (m, 1H, C**H**-OOC-), 4.19 (m, 2H, *J* = 6.5 and 11.5 Hz, -C**H_2_**-OH). ^13^C-NMR (ppm): 14.22 (-CH_3_), 22.78 (-**C**H_2_-CH_3_), 25.00–29.78 (other -CH_2_-), 32.01 (-**C**H_2_-CH_2_-CO), 34.25 (-**C**H_2_-CO), 63.41 (-CH_2_-OH), 70.35 (-CH-OH), 174.48 (-COO-).

### 3.6. Antibacterial and Antifungal Assays of the Synthesized Products

The antibacterial and antifungal assays were carried out in the Microbiology Laboratory of the Faculty of Veterinary Medicine, Universitas Gadjah Mada using the standard method as previously described [23]. *Candida albicans* (*C. albicans*) was used as the representative of fungi, *Staphylococcus aureus* (*S. aureus*) and *Bacillus subtilis* (*B. subtilis*) were used as representatives of gram-positive bacteria, and *Escherichia coli* (*E. coli*) and *Aggregatibacter actinomycetemcomitans* (*A. actinomycetemcomitans*) were used as representatives of gram-negative bacteria.

#### 3.6.1. Antibacterial Activity Assay of Monoacylglycerol

At first, brain heart broth (6.5 g) was dissolved in distilled water (100 mL) and sterilized using an autoclave at 394 K under 15 psi pressure for 15 min. The solution was poured into a petri dish and cooled down to room temperature. The selected bacteria were then introduced separately into the prepared media. The wells for sample injection were formed by using a capillary glass, and thus the well diameters were fixed at 6.0 mm. Each of the synthesized products was dissolved in 20.0% polyethyleneglycol 400 (PEG 400) in distilled water as the diluent at the desired concentration (% *w*/*v*). The sample (50 μL) was added into the wells on the culture media and incubated at 310 K for 30 h. When the sample showed antimicrobial activity, a transparent zone (called the inhibition zone) was formed due to the diffusion process of the sample. The antibacterial activity of the monoacylglycerol was represented from the average diameter inhibition zone (with two replications). The 1.00% 4-isopropyl-3-methylphenol in 20.0% PEG 400 solution was used as the positive control, while the solvent 20.0% PEG 400 was used as the negative control.

#### 3.6.2. Antifungal Activity Assay of Monoacylglycerol

The antifungal activity assay of monoacylglycerol was carried out in a similar procedure to the antibacterial assay, except Sabouraud agar powder, which contained 4.00% of dextrose to replace brain heart broth, was used.

## 4. Conclusions

This research reported the synthesis of 1-monomyristin, 2-monomyristin and 2-monopalmitin through simple and efficient reactions. The chemical structure of the synthesized products was confirmed by FTIR, GC (or LC)-MS, and ^1^H- and ^13^C-NMR spectrometers. These synthesis methods have the potential to be applied in industrial processes owing to the commercially available starting materials, simple process, short time reaction, and high yield. From the antibacterial and antifungal assays, the 2-monopalmitin compound did not show either antibacterial or antifungal activities. Meanwhile, the 1-monomyristin compound exhibited higher antibacterial activity against *S. aureus* and *A. actinomycetemcomitans* and also better antifungal activity against *C. albicans* compared with the positive control. The 2-monomyristin compound performed better for antibacterial activity against *E. coli* than the positive control. These results show a potential application of either 1-monomyristin or 2-monomyristin compounds as antibacterial and antifungal agents in real applications.

## Figures and Tables

**Table 1:** Antibacterial and antifungal assays of 1-monomyristin, 2-monomyristin and 2-monopalmitin against *E. coli*, *S. aureus,* and *C. albicans*.

Sample	Inhibition Zone’s Average (mm)		
	*E. coli*	*S. aureus*	*C. albicans*
0.50% 1-monomyristin	1.5	**10.3**	-
1.00% 1-monomyristin	1.1	5.7	3.5
5.00% 1-monomyristin	4.3	5.9	3.6
10.0% 1-monomyristin	-	**9.1**	2.4
15.0% 1-monomyristin	6.0	**18.9**	4.1
0.25% 2-monomyristin	**34.0**	**23.0**	-
0.50% 2-monomyristin	**29.5**	**20.0**	-
1.00% 2-monomyristin	**22.0**	**13.0**	-
5.00% 2-monomyristin	**21.0**	**13.0**	-
10.0% 2-monomyristin	11.0	5.0	-
0.25% 2-monopalmitin	-	-	-
0.50% 2-monopalmitin	-	-	-
1.00% 2-monopalmitin	-	-	-
5.00% 2-monopalmitin	-	-	-
10.0% 2-monopalmitin	-	-	-
Positive control	12.5	6.6	6.8
Negative control	-	-	-

Negative control: 20.0% PEG 400; Positive control: 1.00% 4-isopropyl-3-methylphenol; -: No activity.

**Table 2:** Antibacterial assays of 1-monomyristin against *B. subtilis* and *A. actinomycetemcomitans*.

Sample	Inhibition Zone Average (mm)	
	*B. subtilis*	*A. actinomycetemcomitans*
0.50% 1-monomyristin	2.4	1.2
1.00% 1-monomyristin	3.6	1.9
5.00% 1-monomyristin	5.7	3.6
10.0% 1-monomyristin	9.2	**7.9**
15.0% 1-monomyristin	12.7	**10.4**
Positive control	16.3	5.5
Negative control	**-**	-

Negative control: 20.0% PEG 400; Positive control: 1.00% 4-isopropyl-3-methylphenol; -: No activity.

## Supplementary Materials

The following are available online.
