# Supplementary material for: Monomyristin and Monopalmitin Derivatives: Synthesis and Evaluation as Potential Antibacterial and Antifungal Agents
Source: Molecules. 2018 Nov 29;23(12):3141. doi: 10.3390/molecules23123141 (PMC6320884; doi:10.3390/molecules23123141)
Supplement: Supplementary file 1 [file molecules-23-03141-s001.pdf]

Supplementary Materials

Monomyristin and Monopalmitin Derivatives: Synthesis and Evaluation as Potential Antibacterial and Antifungal Agents

Jumina [1,*], Asma Nurmala [1], Anggit Fitria [1], Deni Pranowo [1], Eti Nurwening Sholikhah [2], Yehezkiel Steven Kurniawan [1] and Bambang Kuswandi [3]

1. Department of Chemistry, Faculty of Mathematics and Natural Sciences, Universitas Gadjah Mada, Sekip Utara, Yogyakarta 55281, Indonesia
2. Department of Pharmacology and Therapy, Faculty of Medicine, Universitas Gadjah Mada, Sekip Utara, Yogyakarta 55281, Indonesia
3. Faculty of Pharmacy, University of Jember, Jember 68121, Indonesia

* Correspondence: jumina@ugm.ac.id; Tel.: +62 274545188

Received: 8 November 2018; Accepted: date; Published: date

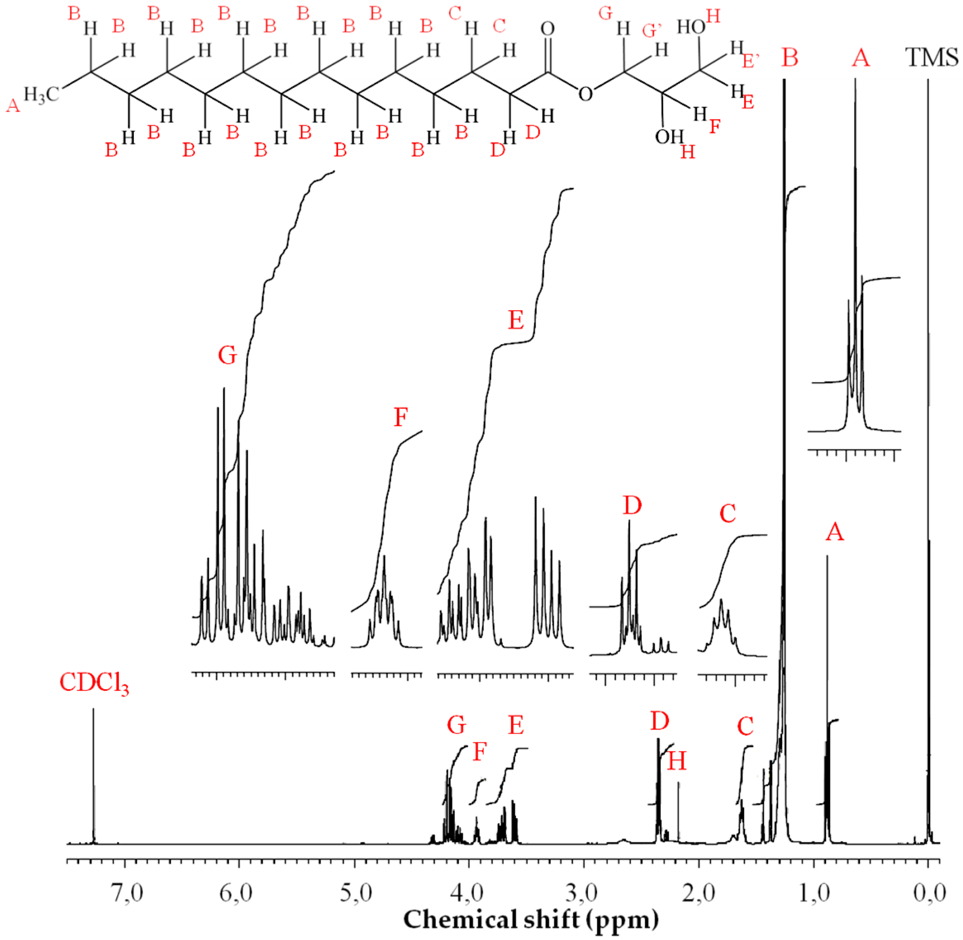

Figure S1. ^{1}H-NMR spectra of 1-monomyristin.

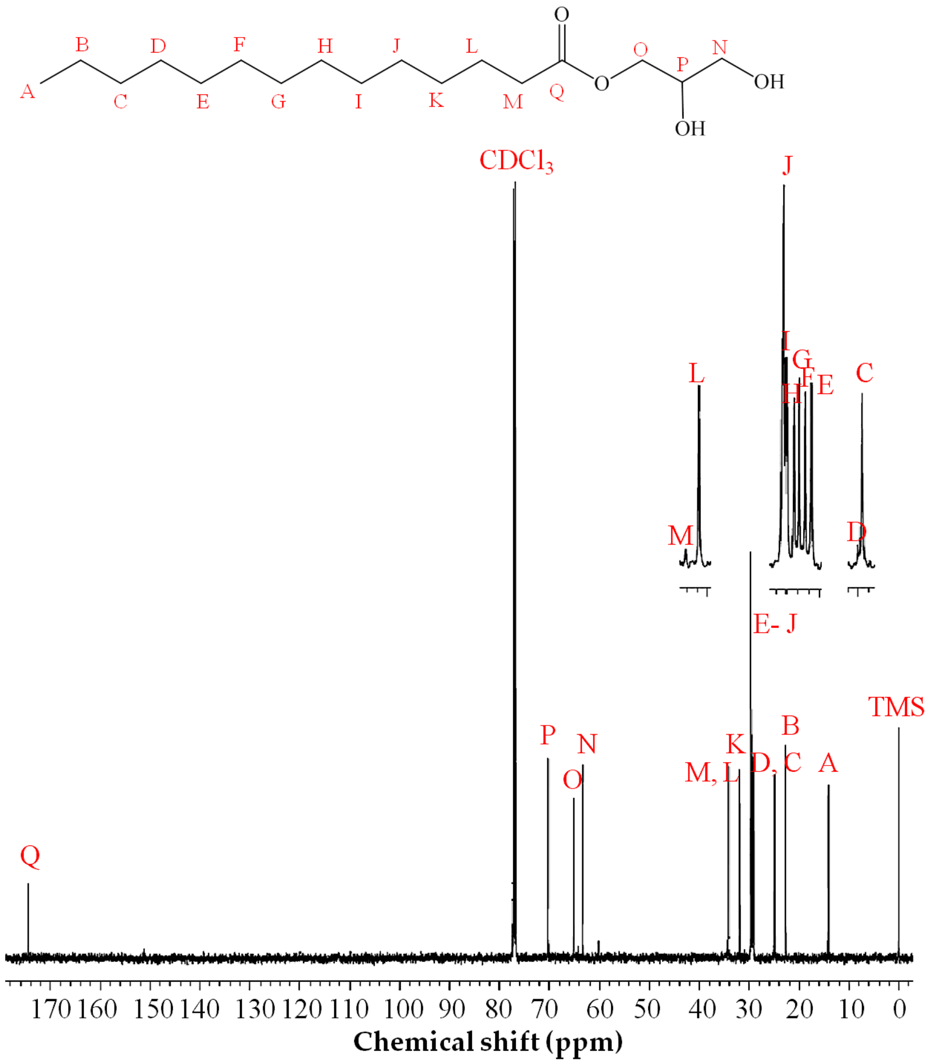

Figure S2. ^{13}C-NMR spectra of 1-monomyristin.

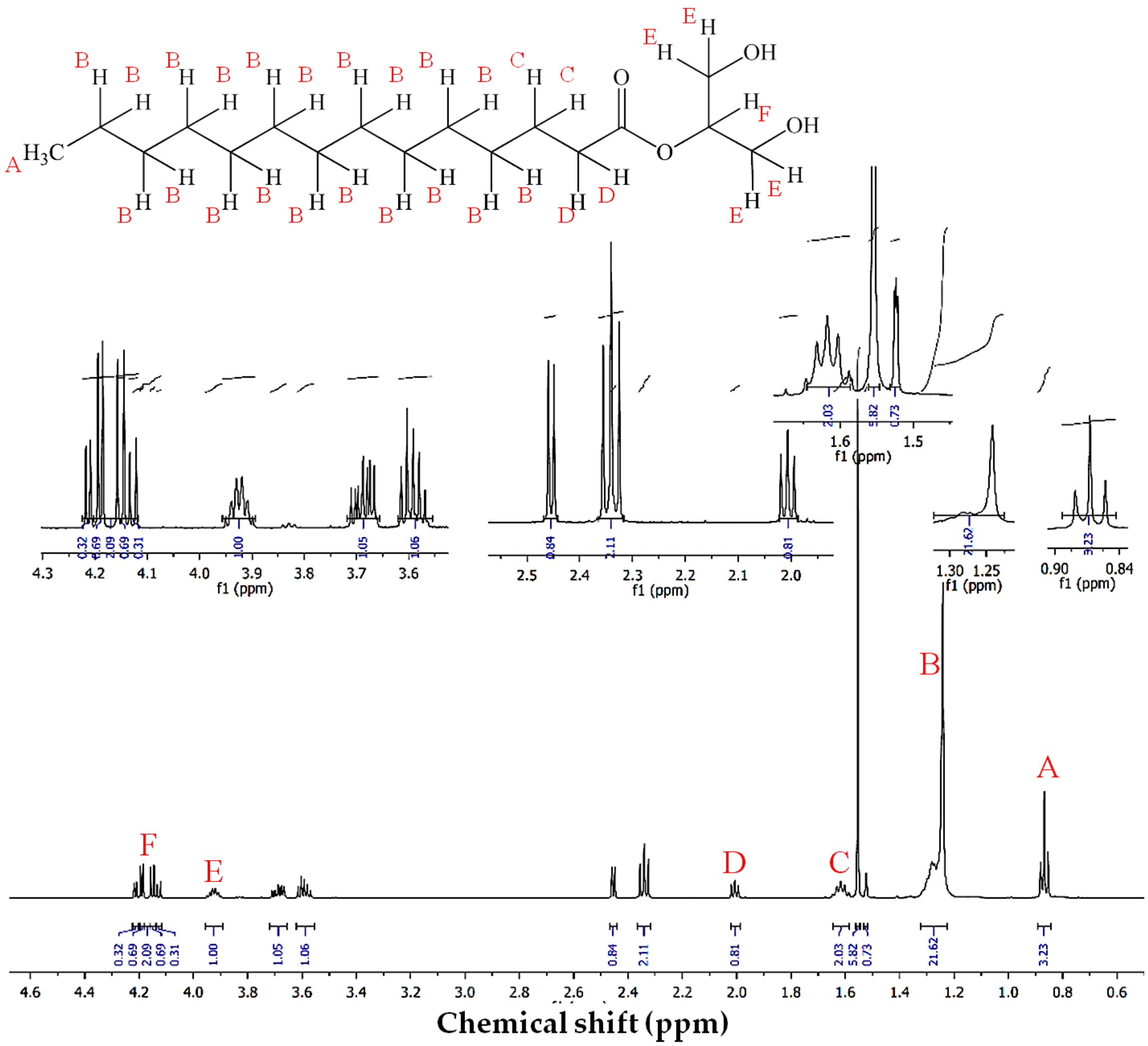

Figure S3. ^{1}H-NMR spectra of 2-monomyristin.

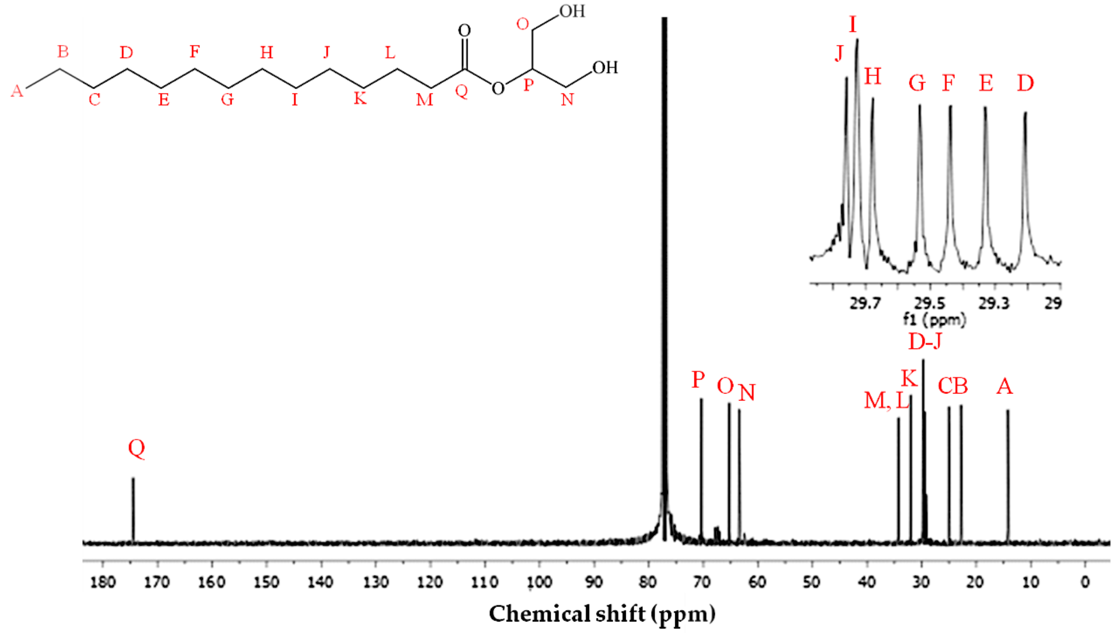

Figure S4. ^{13}C-NMR spectra of 2-monomyristin.

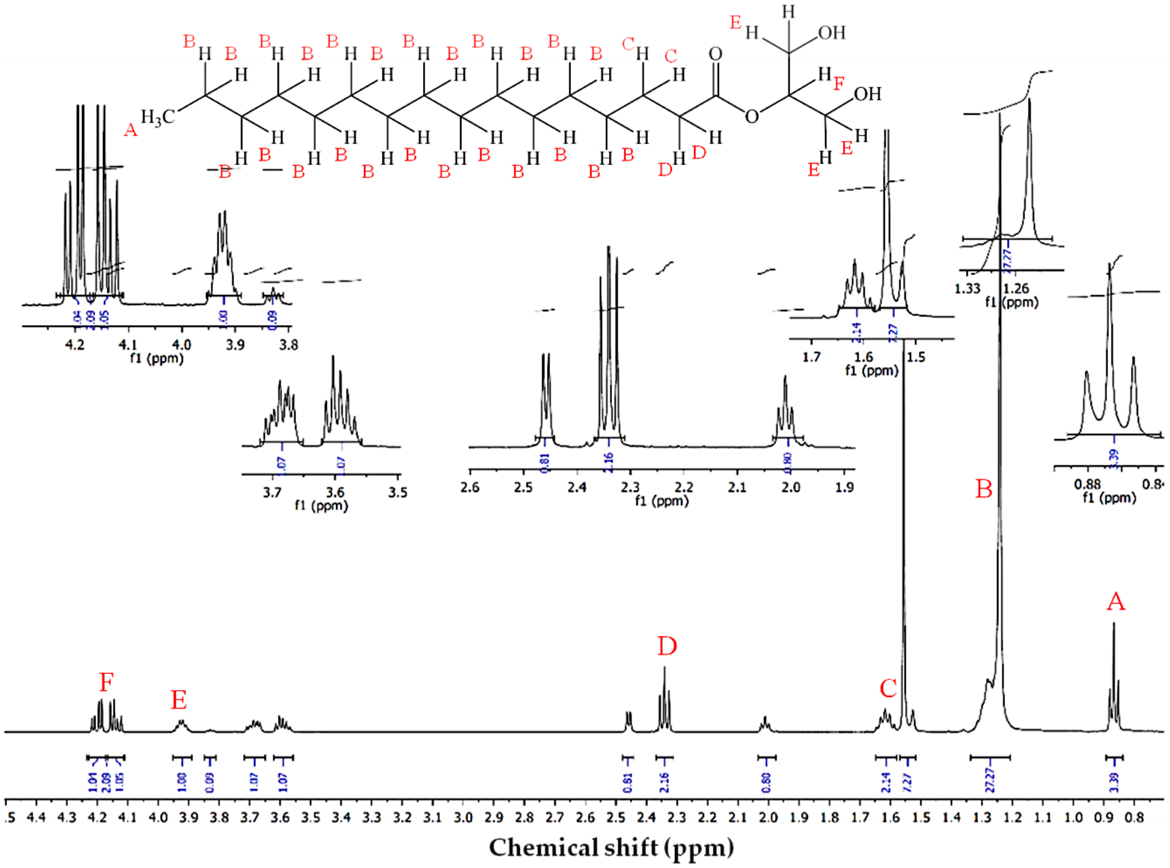

Figure S5. ^{1}H-NMR spectra of 2-monopalmitin.

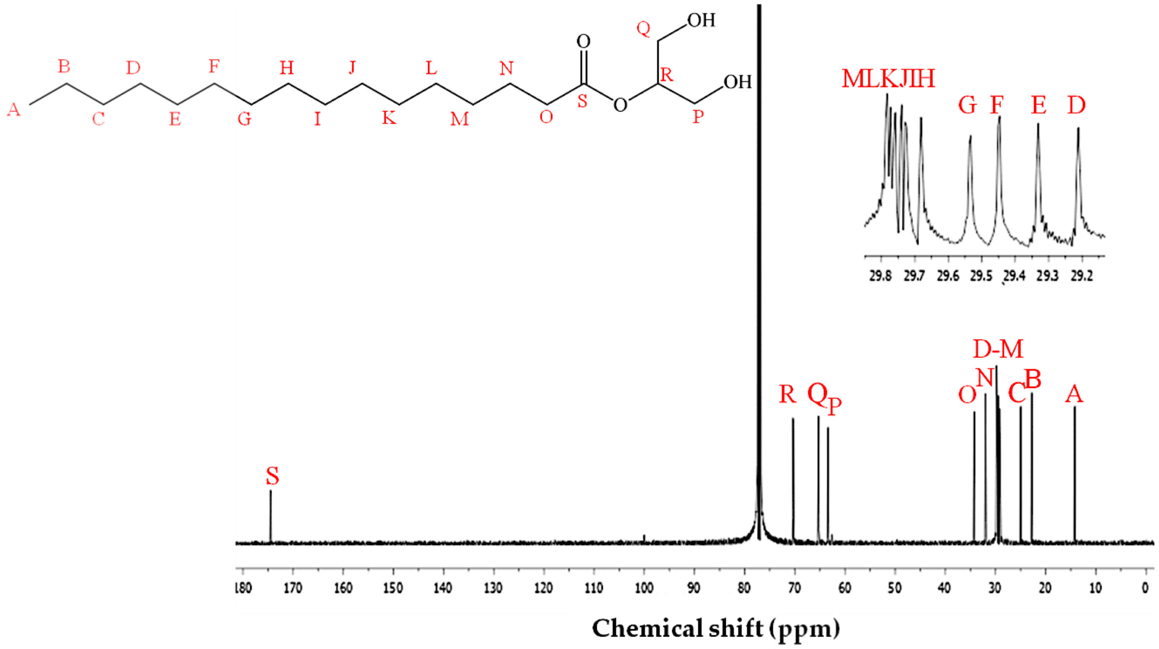

Figure S6. ^{13}C-NMR spectra of 2-monopalmitin.
